# Song for Blood Donor’s Recruitment and Promotion during COVID-19 Pandemic

**DOI:** 10.21315/mjms2022.29.6.17

**Published:** 2022-12-22

**Authors:** Pei Pei Tan, Jernih Abdul Rahman, Mat Noh Sarah, Siti Az-Syazni Amirah Mohamad Azlin, Norsazila Zambree, Sabariah Mohd Noor

**Affiliations:** Transfusion Medicine Department, Hospital Raja Permaisuri Bainun, Perak, Malaysia

**Keywords:** song, blood donor, health promotion, COVID-19

## Abstract

Songs are commonly used in educational public health programmes to facilitate the understanding of health messages. During the COVID-19 pandemic, national blood banks witnessed a pronounced reduction in blood donors. Thus, we created a song with the title ‘Keep the World Beating’ to raise awareness of the need for blood and to promote blood donation. To maximise participation, we carried out a live broadcast of the donation process and the music video on social media, increasing visibility and accessibility to the event. The number of donations increased in the month after the song was released. Songs can be used to strengthen current entertainment-education strategies promoting blood donation and to increase the level of awareness among the local population, thus motivating people to donate blood in a time of need.

## Introduction

Songs have been found to be easily accessible to lay persons, facilitating understanding and providing motivation. A song is also a rapid, cost-effective means of disseminating health messages in public health programmes that encourage behavioural changes and impart key health messages (1, 2).

Entertainment-education, as the name implies, is a type of communication strategy created to reinforce or change attitudes, values, beliefs or social practices by means of integrating educational content into entertainment productions. It is comprised of five elements: i) purposefully curated content; ii) substantiated communication theories; iii) the optimisation of the medium of communication; iv) research with potential audiences before production and v) the collaboration of media practitioners and health professionals as change agents (3).

## Methods

Blood donations are essential to ensure that an adequate supply of blood and blood products is available for transfusions. During the COVID-19 pandemic, blood banks in Malaysia experienced a drop in donations of 40%, leaving them striving to bring in blood donors (4). Because of its proven efficacy, a local blood bank team in a tertiary referral hospital wrote and produced a song to promote blood donations. Meetings were organised with blood transfusion experts, literature and song searches were performed and the song lyrics were written to include key messages about the importance of blood donation and voluntary blood donors. Awareness words and phrases, such as ‘donate blood’, ‘save lives’ and ‘blood is precious’ and motivating words, such as ‘hero’, ‘hopes’ and ‘sacrifice’ were used to emphasise the importance of blood donors.

The melody was composed by a blood bank officer. The song was culturally appropriate, and the melody was catchy, repetitive and easily memorable. In collaboration with a professional music producer, the song was recorded in a music studio by blood bank staff, which included a public relations officer, nurses, medical officers and blood transfusion specialists. It was given the title ‘Keep the World Beating’ in line with the recent World Blood Donor Day (WBDD) campaign. Given the multiracial composition of the local population and to ensure acceptance and cultural appropriateness, the song was made in English and the Malay language.

## Results

We incorporated the song that we created into the WBDD celebration, which was intended to raise public awareness of the importance of regular and voluntary blood donation to maintain the balance between demand and supply (5). A live broadcast of the donation process and the music video was done on social media (Facebook and FM radio) to increase visibility and accessibility. Since its release, the musical video has achieved 280 likes, 8,000 views and 142 shares on Facebook (6).

The feedback has been largely positive, and blood donors expressed feeling joy and appreciated while learning the importance of voluntary blood donation. We believe this may increase donors’ satisfaction and retention (7). The number of donations the month before the song was released was 2,110; this increased to 2,419 donations in the month after the song’s release.

## Discussion

While our findings are not enough to prove the value of a song in increasing donor recruitment or sustainability, our results suggest that this is an area worth exploring in future trials. The planning and execution of the song were done in line with the five elements of the entertainment-education communication strategy. The current worldwide trend of the powerful impact of social media on daily lives (8) suggests that entertainment education on social media can be used to further the promotion of blood donation by increasing the level of awareness among the local population.

## Conclusion

In conclusion, we believe that creative content creation—in this case, a song—can motivate people to donate blood in a time of need.
